# Childhood loneliness as a specific risk factor for adult psychiatric disorders

**DOI:** 10.1017/S0033291721001422

**Published:** 2023-01

**Authors:** Yllza Xerxa, Leslie A. Rescorla, Lilly Shanahan, Henning Tiemeier, William E. Copeland

**Affiliations:** 1 Department of Child and Adolescent Psychiatry, Erasmus University Medical Center, Rotterdam, the Netherlands; 2 Department of Psychiatry, Vermont Center for Children, Youth and Families, University of Vermont, Burlington, USA; 3 Department of Psychology, Bryn Mawr College, Bryn Mawr, PA, USA; 4 Department of Psychology, Jacobs Center for Productive Youth Development, University of Zürich, Zürich, Switzerland; 5 Department of Social and Behavioral Sciences, Harvard TH Chan School of Public Health, Boston, USA

**Keywords:** Adulthood anxiety and depression, childhood, lonely children, repeated assessment

## Abstract

**Background:**

Loneliness is a major risk factor for both psychological disturbance and poor health outcomes in adults. This study aimed to assess whether *childhood* loneliness is associated with a long-term disruption in mental health that extends into adulthood.

**Methods:**

This study is based on the longitudinal, community-representative Great Smoky Mountains Study of 1420 participants. Participants were assessed with the structured Child and Adolescent Psychiatric Assessment interview up to eight times in childhood (ages 9–16; 6674 observations; 1993–2000) for childhood loneliness, associated psychiatric comorbidities and childhood adversities. Participants were followed up four times in adulthood (ages 19, 21, 25, and 30; 4556 observations of 1334 participants; 1999–2015) with the structured Young Adult Psychiatric Assessment Interview for psychiatric anxiety, depression, and substance use outcomes.

**Results:**

Both self and parent-reported childhood loneliness were associated with adult self-reported anxiety and depressive outcomes. The associations remained significant when childhood adversities and psychiatric comorbidities were accounted for. There was no evidence for an association of childhood loneliness with adult substance use disorders. More associations were found between childhood loneliness and adult psychiatric symptoms than with adult diagnostic status.

**Conclusion:**

Childhood loneliness is associated with anxiety and depressive disorders in young adults, suggesting that loneliness – even in childhood – might have long-term costs in terms of mental health. This study underscores the importance of intervening early to prevent loneliness and its sequelae over time.

## Introduction

Loneliness is a distressing emotional state that arises from the discrepancy between one's perceived and desired levels of social connection (Perlman & Peplau, 1981). As such, perceived loneliness can exist for individuals who are not socially isolated (Cacioppo et al., 2006). Loneliness is a major risk factor for psychological disturbance and poor health outcomes in adulthood. In population-based studies, loneliness in adolescents has been prospectively associated with social anxiety (Maes et al., 2019; Teo, Lerrigo, & Rogers, 2013) and depression (Qualter, Brown, Munn, & Rotenberg, 2010).

Loneliness is relatively common among both children and adults. For example, between 11% and 27% of children (aged 10–15 years) reported feeling lonely in the UK (Snape & Manclossi, 2018; Yang, Petersen, & Qualter, 2020), whereas more than 40% of Americans of all ages reported feeling lonely (Cacioppo, Grippo, London, Goossens, & Cacioppo, 2015). Perceived loneliness is also associated with a substantial increase in the risk of premature mortality, independent of income, education, sex, and ethnicity (Cacioppo, Cacioppo, Capitanio, & Cole, 2015; Holt-Lunstad & Smith, 2016; Miller, 2011). A longitudinal analysis of four nationally representative US samples showed the influence of social isolation on several biomarkers of cardiovascular heart disease including hypertension, body mass index, and waist circumference and inflammation (High-sensitivity C-reactive protein) across the lifespan (Yang et al., 2016). The magnitude of this effect is comparable to that of smoking and obesity or physical inactivity.

Much less is known about the long-term effects of *childhood* loneliness. A recent meta-analysis of 102 cross-sectional studies of childhood loneliness found associations with social anxiety (*M*_age_ below 21 years) (Maes et al., 2019). The same meta-analysis looked at 10 longitudinal studies and reported small but positive associations between loneliness and social anxiety symptoms both within and across time, and across childhood and adolescence. To our knowledge, there is one longitudinal study on the association between childhood loneliness and depression in pre-school and school-aged children (Qualter et al., 2010). No studies to date have addressed the key question of whether childhood loneliness is associated with a long-term disruption in mental health that extends into adulthood.

Parents and children often disagree about the presence and severity of child symptoms and psychopathology (Achenbach, McConaughy, & Howell, 1987; De Los Reyes et al., 2011). Thus, parental and child ratings may capture overlapping but largely distinct information about a child's experience of loneliness and associated risk for psychiatric disorder outcomes. Loneliness is, by definition, a subjective, internal state of mind. Yet, it is reasonable to expect that raters who know the child well (e.g. parents or friends) may have access to information about the individual's loneliness and therefore provide accurate judgments of internal traits.

To address these gaps, we conducted a large population-based study of the association between childhood loneliness and psychiatric disorders in adulthood. We focused on loneliness measures assessed eight times from children and a parent from ages 9 to 16 years. Participants were then followed up four times in adulthood between ages 19 and 30 years, and assessed for anxiety, depression, and substance use symptoms and disorders. The present study had three main aims. First, we aimed to examine whether repeated measures of loneliness in childhood are associated with a broad range of psychiatric problems that include anxiety, depression, and substance use disorders in adults. We also examined the associations between the groups of childhood loneliness trajectories and these psychiatric outcomes. Second, we aimed to examine whether the associations between childhood loneliness and adult psychiatric disorders persist after accounting for other co-occurring adverse childhood experiences. Finally, we examined whether parent-reported loneliness predicts adult outcomes similar to child-reported loneliness.

## Method

### Participants

**Fig. 1.**
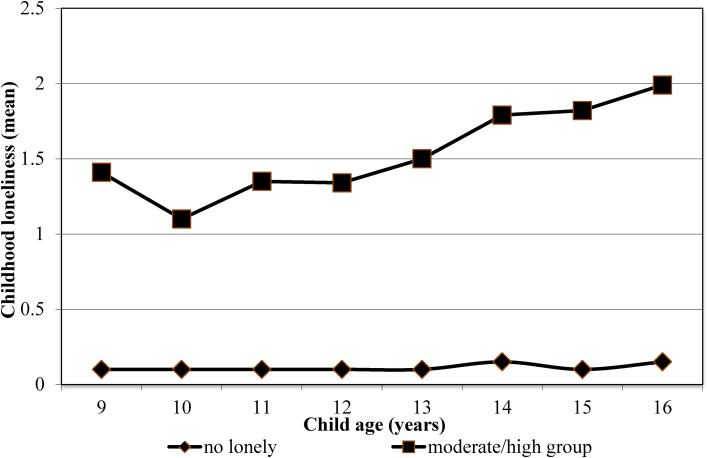
Childhood loneliness trajectories.

This report follows the Strengthening the Reporting of Observational Studies in Epidemiology (STROBE) reporting guideline for cohort studies (Von Elm et al., 2007). Data were drawn from the Great Smoky Mountains Study (GSMS), a longitudinal, community-representative cohort in which participants were followed up from age 9 years into adulthood. The GSMS, which enrolled children in 11 predominantly rural North Carolina counties, was originally designed to estimate the prevalence of mental illness and service (Costello, Mustillo, Erkanli, Keeler, & Angold, 2003). Initially, three cohorts of children, aged 9, 11, and 13 years, were recruited from a pool of approximately **12 000 children using a two-stage sampling design, resulting in 1420 participants [630 girls (weighted percentage, 49.0%)] (Costello et al., 2003). Sampling weights were applied to adjust for differential probability of selection. An ascertainment figure appears in Fig. 1 in the online Supplementary material, and the original study articles (Copeland, Angold, Shanahan, & Costello, 2014; Costello et al., 1996, 2003) provide additional detail on sampling and derivation of sample weights.

Annual assessments were completed until participants were 16 years old and then they were assessed again at ages 19, 21, 25, and 30 years, for a total of 11 230 total assessments from January 1993 to December 2015. Across all assessments, 83.0% of possible interviews (11 230 of 13 530) were completed. Race/ethnicity was determined based on parent report. Before all interviews, parents and children signed institutional review board-approved informed consent and assent forms. All procedures and protocols for the present study were approved by the Duke University Institutional Review Board.

### Measures

#### Childhood loneliness

Questions about perceived loneliness were collected as part of self or parental-report interviews using the Child and Adolescent Psychiatric Assessment (CAPA) from ages 9 to 16 (Angold & Costello, 2000). The CAPA defines loneliness as ‘a feeling of being alone and/or friendless, regardless of the justification for the feeling; total daily duration of at least 1 h’. The CAPA focuses on the 3 months immediately preceding the interview as its primary period. To assess this item, participants and their parents were asked first ‘Do you think s/he feels lonely?’ and then ‘Sometimes children feel that they have no one who would help them. Does s/he ever feel like that?’. These two questions are followed by a series of secondary prompts, if necessary, to clarify whether the child met the operational definition of lonely. Secondary prompts comprised of items such as: ‘How often is that?’, ‘When was the last time?’, ‘Does s/he feel cared for by friends?’, ‘Does s/he feel lonely even though s/he has some friends?’. More details on how loneliness is defined and assessed through primary and follow-up prompts can be found in online Supplementary Fig. 2.

These items index both a perceived quantitative lack of contacts in one's social network and a perceived qualitative deficit in existing relations. As such, this construct is best considered perceived social isolation (feeling lonely) as opposed to objective social isolation (being lonely). Different dimensions of social functioning (e.g. subjective loneliness, network quality, and network size) have been found to have different associations with health (Victor, Scambler, Bond, & Bowling, 2000). Subjective loneliness has been linked to psychological consequences e.g. Weiss (1973) and Peplau and Perlman (1979). Other studies of loneliness have used a similar construct (Qualter et al., 2010, 2013).

Loneliness is scored on a three-point rating scale 0 = ‘not at all’, 1 = ‘the interviewee definitely feels lonely in a way that interferes with at least two activities and is uncontrollable’, and 2 = ‘the interviewee feels lonely most of the time’. For the current study, loneliness was scored dichotomously (‘0’ *v.* ‘1’ and ‘2’) and we aggregated the loneliness measure between ages 9 and 16 into a single measure of childhood loneliness.

#### Adult psychiatric disorders and functioning

All outcomes except where noted were assessed using the Young Adult Psychiatric Assessment (Angold et al., 1999), an upward extension of the CAPA interview administered to the participants. The assessment of adult psychiatric disorders resembled that of childhood disorders, but with only self- (and not parent-) reports. A 3-month ‘primary period’ was selected because longer recall periods are associated with forgetting and recall bias (Copeland et al., 2014; Costello et al., 1996, 2003). Disorders included any DSM generalized anxiety disorder, social phobia, post-traumatic stress disorder, panic disorder, agoraphobia, depressive disorder, and substance use disorder (including, nicotine, alcohol, cannabis, and other illicit drugs). For the current study, we examined anxiety, depression, as well as substance use disorders. Each psychiatric disorder measure between ages 19 and 30 is aggregated into a single measure of adult psychiatric status. The participant was positive for diagnosis if criteria were met at any adult observation. In addition, we examined symptoms scores of the total anxiety, depressive, and substance use symptoms.

#### Covariates

Parent and child characteristics examined as potential confounders are depicted in the Appendix in the online Supplementary material. These included the following: sex of the child, rural *v.* urban area, family hardships (including low socio-economic status, single parent, change in parent structure, maltreatment, and depression of the mother), and childhood psychiatric comorbidities (including anxiety, depression, and disruptive behavior). For psychiatric symptoms, the CAPA focuses on the 3 months immediately preceding the interview to minimize recall bias. Scoring programs written in SAS statistical software (SAS Institute Inc.) combine information about the date of onset, duration, and intensity of each symptom to create DSM diagnoses. Test–retest reliability and validity of the CAPA diagnoses are similar to other psychiatric interviews (Angold & Costello, 1995, 2000). Psychiatric disorders assessed included anxiety disorders, depression disorder, and substance use disorders. The categories of family hardships or childhood adversities were assessed at each observation. A full description of these variables is available in a previous publication (Copeland, Wolke, Angold, & Costello, 2013). In our study, we aggregated childhood covariates between ages 9 and 16 into a single measure of each covariate.

### Statistical analysis

First, we computed descriptive statistics for self- and parent-reported loneliness at different time points. Then, we tested prospective associations of childhood loneliness (between ages 9 and 16 years) reported by parent and child with adult anxiety, depression, and substance use diagnostic status (between ages 19 and 30 years) with separate weighted logistic regressions. Then, we tested prospective associations of childhood loneliness with adult anxiety, depressive, and substance use symptom scores with separate linear regressions. All analyses applied sampling weights; therefore, results provide estimates of the original representative population from which the sample was drawn.

As a follow-up to primary analysis, a latent class growth analysis was used to test for the associations of childhood loneliness trajectories with adult psychiatric disorders. Trajectories of loneliness were modeled in children from whom data were available for two or more time points (*N* = 1334). Models with two to five trajectories were assessed and compared. We used the full information maximum likelihood ratio to account for missing data. To assess model fit, we evaluated the bootstrap likelihood ratio test, the Bayesian information criterion, and the Akaike information criterion (Nylund et al., 2007). Subsequently, linear regression analyses were used to assess childhood loneliness trajectory in relation to adult outcomes.

Across all assessments, 83% of possible interviews were completed (online Supplementary Table S1). All 1420 participants were interviewed at least once in childhood (ages 9–16); 1260 participants (88.7%) had 3+ childhood observations. Of the total sample, 1334 (94.0%) were followed up at least once in adulthood at ages 19, 21, 25, or 30. All analyses were performed using SAS 9.4 software. All missing values of the potential confounding factors were imputed using multiple imputations. With the Markov Chain Monte Carlo multiple imputation techniques, 10 complete data sets were created. Multivariate analyses were performed on each imputed data set, and effect estimates were pooled (Schunk, 2008).

## Results

### Prevalence of loneliness and the associations with sociodemographic factors

Descriptive information is provided in Table 1. The prevalence of childhood/adolescent loneliness was 13.4%, meaning that >1 in 10 children reported feeling lonely at some point by age 16. Overall, the prevalence of loneliness differed by sex of the child (more common in girls than boys), but not by race/ethnicity and rural *v.* urban area. In our study, the within-individual correlation of loneliness measure over time was *r* = 0.42. Concurrent analyses showed that childhood loneliness was associated with a change in parent structure, as well as maternal depression, after adjustment for sex of the child. We observed no associations between childhood loneliness and low socio-economic status, single-parent family status, and child maltreatment.

**Table 1.** Prevalence of childhood loneliness between ages 9 and 16 and association with childhood adversities

	Never lonely *N* (%)	Ever lonely *N* (%)	*p* value^a^
Overall childhood loneliness	1230 (86.6)	190 (13.4)	
Sex, girl	529 (43)	101 (53.2)	0.042
Rural areas	739 (60.1)	100 (52.6)	0.863
Psychosocial risk			
Low socioeconomic status	489 (39.8)	84 (44.2)	0.131
Single parent	491 (39.9)	105 (44.7)	0.256
Change in parent structure	386 (31.4)	76 (40)	0.305
Maltreatment	262 (21.3)	74 (38.9)	0.136
Depression mother	193 (15.7)	64 (33.7)	0.001

Numbers denote children included in one or more analyses. Numbers are unweighted, and percentages are weighted.

a *p* value from binary logistic regression of childhood loneliness and childhood adversities outcome. The models [ORs] are adjusted for child sex.

Next, we tested the associations of child- and parent-reported loneliness with anxiety, depression, and substance use disorder within childhood. Child- and parent-reported loneliness was concurrently associated with childhood anxiety, depression, and substance use disorder (Table 2). The association of loneliness with psychiatric disorder outcomes was consistently stronger if loneliness was rated by the child than by the parent.

**Table 2.** Prevalence of childhood loneliness between ages 9 and 16 and association with childhood psychiatric disorders

	Child-report loneliness		
	Never lonely *N* (%)	Ever lonely *N* (%)	*p* value^a^
Overall childhood loneliness	1342 (94.5)	78 (5.5)	
Psychiatric disorders			
Any anxiety diagnosis	86 (6.4)	22 (28.2)	<0.001
Any depression diagnosis	28 (2.1)	18 (23.1)	<0.001
Disruptive behavior disorder	122 (9.1)	19 (24.4)	0.001
Psychiatric symptoms	*M* (s.d.)	*M* (s.d.)	
Any anxiety symptoms	1.7 (2.2)	4.9 (3.9)	<0.001
Any depression symptoms	0.2 (0.1)	0.2 (0.4)	<0.001
Disruptive behavior	0.1 (0.3)	0.2 (0.4)	<0.001
Parent-report loneliness			
Psychiatric disorders	1294 (91.1)	126 (8.9)	
Any anxiety diagnosis	82 (6.1)	10 (12.8)	<0.001
Any depression diagnosis	59 (4.4)	11 (14.1)	<0.001
Disruptive behavior disorder	229 (1.1)	22 (28.2)	<0.001
Psychiatric symptoms	*M* (s.d.)	*M* (s.d.)	
Any anxiety symptoms	1.7 (2.3)	5.4 (4.6)	<0.001
Any depression symptoms	1.4 (1.3)	3.3 (2.0)	<0.001
Disruptive behavior	2.7 (2.9)	5.3 (4.2)	<0.001

Numbers denote children included in one or more analyses. Values are frequencies for categorical (numbers are unweighted, and percentages are weighted). Means and standard deviations (*M* ± s.d.) for continuous measures.

a *p* value from binary logistic regression of childhood loneliness and childhood psychiatric disorder outcomes. The models [ORs] are adjusted for child sex and adversities.

Furthermore, we tested whether childhood loneliness was associated with the number of arguments with peers and teased or bullied during childhood (online Supplementary Table S1). Concurrent associations showed that childhood loneliness was associated with the number of arguments with peers and teased or bullied in childhood. In contrast, we observed no associations of childhood loneliness and frequency of contact with peers, confidante with family and peers, as well as shyness with peers. These associations and non-associations suggest that our measure best approximates subjective loneliness.

### Childhood loneliness and adult psychiatric disorders

We then examined the associations of child- and parent-report loneliness and psychiatric disorders in adulthood adjusted for demographic variables and childhood adversities. As shown in Table 3, self-reported loneliness was associated with an anxiety disorder ( Odds ratio (OR) = 3.53, 95% Confidence interval (CI) 1.55–8.04, *p* = 0.002) but not with depressive and substance use disorders. Next, to test whether childhood loneliness was associated with adult psychiatric symptoms. Self-reported loneliness was associated with anxiety (*B* = 1.20, 95% CI 0.43–1.97, *p* = 0.002), and depressive symptoms scores (*B* = 0.76, 95% CI 0.27–1.25, *p* = 0.002), but not with substance use symptoms (*B* = 0.20, 95% CI, **−**0.14 to 0.54, *p* = 0.246). Effect estimates were modestly attenuated when we accounted for childhood psychiatric status (model 2).

**Table 3.** The association between childhood loneliness between ages 9 and 16 with adult psychiatric and substance use disorders

			Psychiatric disorders					
			Any anxiety diagnosis		Any depression diagnosis		Substance use disorder	
		Overall	OR (95% CI)	*p* value	OR (95% CI)	*p* value	OR (95% CI)	*p* value
Child-report loneliness	Never lonely *n* (%)	1260 (94.4)	184 (14.6)		137 (10.9)		398 (31.6)	
	Ever lonely *n* (%)	74 (5.6)	33 (44.6)		20 (27.0)		34 (45.9)	
Model 1			5.95 (2.77–12.7)<style for 95% CI values>	<0.0001	2.72 (1.08–6.83)	0.033	1.95 (0.95–3.97)	0.067
Model 2			3.53 (1.55–8.04)	0.002	1.86 (0.73–4.71)	0.190	1.65 (0.73–3.74)	0.227
Parent-report loneliness	Never lonely *n* (%)	1215 (91.1)	177 (14.6)		136 (11.2)		392 (32.3)	
	Ever lonely *n* (%)	119 (8.9)	40 (33.6)		21 (17.6)		40 (33.6)	
Model 1			2.95 (1.51–5.75)	0.001	1.61 (0.61–4.23)	0.331	1.25 (0.62–2.54)	0.530
Model 2			1.43 (0.67–3.04)	0.354	0.91 (0.32–2.57)	0.861	0.94 (0.42–2.12)	0.887
			Psychiatric symptoms					
			Any anxiety symptoms		Any depression symptoms		Substance use symptoms	
			*B* (95% CI)	*p* value	*B* (95% CI)	*p* value	*B* (95% CI)	*p* value
Child-report loneliness	Never lonely, *M* (s.d.)		2.5 (3.2)		2.1 (2.0)		1.1 (1.4)	
	Ever lonely, *M* (s.d.)		4.5 (4.2)		3.4 (2.6)		1.5 (1.7)	
Model 1			1.91 (1.16–2.66)	<0.0001	1.25 (0.76–1.72)	<0.0001	0.38 (0.06–0.72)	0.020
Model 2^a^			1.20 (0.43–1.97)	0.002	0.76 (0.27–1.25)	0.002	0.20 (−0.14 to 0.54)	0.246
Parent-report loneliness	Never lonely, *M* (s.d.)		2.4 (3.2)		2.1 (2.0)		1.1 (1.4)	
	Ever lonely, *M* (s.d.)		4.4 (4.1)		3.2 (2.4)		1.3 (1.5)	
Model 1			1.78 (1.17–2.40)	<0.0001	0.93 (0.54–1.32)	<0.0001	0.09 (−0.17 to 0.37)	0.478
Model 2^a^			1.12 (0.49–1.75)	0.001	0.48 (0.08–0.88)	0.019	−0.06 (−0.33 to 0.22)	0.684

Regression analysis of childhood loneliness and adult psychiatric outcomes. Values are frequencies for categorical (numbers are unweighted, and percentages are weighted) and means and standard deviations (*M* ± s.d.) for continuous measures. Model 1 is adjusted for child sex and childhood adversities. Childhood adversities include low family socioeconomic status, change in parent structure, mother depression. Model 2 is additionally adjusted for childhood psychiatric disorders. Child psychiatric disorders include anxiety, depression, and disruptive disorders.

OR indicate binary logistic regression coefficients for psychiatric disorders.

*B* statistics indicate linear regression coefficients for psychiatric symptoms.

a Model 2 is additionally adjusted for childhood psychiatric symptoms.

In fully adjusted models (model 2), we observed no associations between parent-reported loneliness and any measure of adult diagnostic status. Parent-reported loneliness was, however, associated with adult anxiety (*B* = 1.12, 95% CI 0.49–1.75, *p* = 0.001) and depressive (*B* = 1.12, 95% CI 0.49–1.75, *p* = 0.001) psychiatric *symptoms*.

### Childhood loneliness trajectories and adult psychiatric disorders

We tested associations of childhood loneliness trajectories with adult psychiatric disorders and symptoms. Figure 1 illustrates the mean scores for the total sample of the three trajectory groups. The first class (*N* = 1260, 94.4%), termed ‘low’, consisted of children who reported no or very few feelings of loneliness over time. The second class (*N* = 65, 5.1%), labeled ‘moderate’ included children with a moderate increasing level of loneliness. Finally, there was a small group of children (*N* = 9, 0.5%), with slightly higher levels of lonely feelings than the moderate group. The three-class model was found to be the optimal model, with the lowest Bayesian information criterion scores and *p* values = 0.05 for the bootstrap likelihood ratio test (online Supplementary Table S2). Because of the sample size for the high group, it will be combined with the moderate group for all analyses.

As shown in Table 4, children in the moderate/high groups had higher levels of anxiety disorders than the children who were in the low group. There were no differences in depression and substance use disorder between groups. Children in the moderate/high groups had higher levels of anxiety and depressive symptoms than those in the low loneliness group. No differences in substance use symptoms between groups were found.

**Table 4.** Childhood loneliness trajectories with adult psychiatric and substance use disorders

	Psychiatric disorders					
	Any anxiety diagnosis		Any depression diagnosis		Substance use disorder	
Child-report loneliness	OR (95% CI)	*p* value	OR (95% CI)	*p* value	OR (95% CI)	*p* value
Model 1						
No lonely	Ref		Ref		Ref	
Moderate/high group	6.34 (2.78–9.94)	<0.0001	3.71 (1.09–7.01)	0.010	2.75 (0.91–4.52)	0.040
Model 2						
No lonely	Ref		Ref		Ref	Ref
Moderate/ high group	3.82 (1.45–7.24)	0.001	1.34 (0.72–4.99)	0.193	1.89 (0.60–3.54)	0.127
	Psychiatric symptoms					
	Any anxiety symptoms		Any depression symptoms		Substance use symptoms	
Child-report loneliness	*B* (95% CI)	*p value*	*B* (95% CI)	*p value*	*B* (95% CI)	*p value*
Model 1						
No lonely	Ref		Ref		Ref	
Moderate/ high group	2.65 (1.21–2.76)	<0.0001	2.34 (0.70–1.82)	<0.0001	0.81 (0.05–0.85)	0.010
Model 2^a^						
No lonely	Ref		Ref		Ref	
Moderate/high group	2.31 (0.55–2.05)	0.001	1.68 (0.21–1.41)	0.001	0.43 (−0.10 to 0.73)	0.212

Regression analysis of childhood loneliness trajectories and adult psychiatric outcomes. Model 1 is adjusted for child sex and childhood adversities. Childhood adversities include low family socioeconomic status, change in parent structure, mother depression. Model 2 is additionally adjusted for childhood psychiatric disorders. Child psychiatric disorders include anxiety, depression, and disruptive disorders.

Odds ratio (OR) indicate binary logistic regression coefficients for psychiatric disorders.

*B* statistics indicate linear regression coefficients for psychiatric symptoms.

a Model 2 is additionally adjusted for childhood psychiatric symptoms.

## Discussion

This prospective population-based study examined the associations of childhood loneliness and adult psychiatric disorders while carefully controlling other common childhood adversities and childhood psychiatric functioning. We highlight three key findings. First, childhood loneliness was prospectively associated with adult self-reported anxiety and depression but not substance use outcomes. Moreover, children exposed to persistently moderate and high levels of loneliness trajectories showed more symptoms of anxiety and depression. Second, these associations remained when we account for childhood-assessed adversities and psychiatric comorbidities (e.g. anxiety, depression, and conduct disorder). Third, the associations were stronger for self- than for parent-reported childhood loneliness, although there was evidence of cross-informant associations for psychiatric symptoms. Overall, our findings suggest that children's experience of loneliness is associated with increased risk for psychiatric disorders, and has the potential to have lifelong effects on one's social and emotional functioning.

Prior studies examining developmental trajectories of loneliness from childhood to early adulthood have indicated that between 3% and 22% of people experience persistent loneliness (Qualter et al., 2010, 2013; Vanhalst, Luyckx, & Goossens, 2014; Yang et al., 2020). With our data spanning 20+ years and multiple informants, we were able to extend previous findings across developmental periods indicating that loneliness experienced in childhood had particularly robust associations with adult self-reported anxiety and depression. Importantly, our results suggest that the associations of childhood loneliness with adult psychiatric outcomes were independent of childhood sociodemographic factors, adversities, and psychiatric functioning, indicating a unique contribution of loneliness to mental health outcomes later in life. No such associations were observed for adult substance disorders or symptoms.

The trajectory analysis largely confirmed the primary analysis with evidence that moderate to high loneliness is likely to affect anxiety and depression outcomes. Within these groups, loneliness tended to peak between ages 14 and 16 during early adolescence when peer groups and influences are taking on increasing importance. At the same time, it is notable that the rank order of the trajectories remained the same from childhood through adolescence suggesting the potential for early identification of children at risk. These results suggest that childhood loneliness may be a potential risk factor (Offord & Kraemer, 2000) to emerging mental health problems and that age-appropriate interventions for loneliness may alleviate later suffering.

The finding that early childhood loneliness precedes mental health outcomes suggests that loneliness may be attributed to myriad processes including neural (Cacioppo, Capitanio, & Cacioppo, 2014), neuroendocrine (Cacioppo et al., 2015), and genetic mechanisms (Goossens et al., 2015), as well as physiological stress state. The evolutionary theory of loneliness posits that loneliness increases the motivation to attend to and approach social stimuli for potential relief from the aversive state (e.g. like hunger, thirst, and pain promotes behavior change to increase the likelihood of the survival of one's genes) (Boomsma, Cacioppo, Muthén, Asparouhov, & Clark, 2007). The evolutionary theory of loneliness suggests that such experiences may contribute to (a) increased vigilance for social threats along with increased anxiety, hostility, and social withdrawal to avoid predation, (b) increased sleep fragmentation, (c) elevated vascular activity, increase extended periods of hypothalamo–pituitary–adrenal activations, and decreased gene expression and immunity to deal with potential assaults that may arise, (d) decreased impulse control (e.g. prepotent responding), and (e) increased depressive symptomatology (Cacioppo et al., 2014). Tests of these mechanisms are beyond the scope of this study but may explain the long-term associations observed here.

Our study extended prior findings by using a multi-informant approach to address the potential for a bias when relying only on self-report data. Because loneliness is a subjective experience, and an internal state of mind, it is often examined using self-reports (Heinrich & Gullone, 2006). However, as close others (e.g. parents, teacher, or friends) can observe behavioral changes resulting from loneliness, they could provide additional information on children's loneliness (Cacioppo, Cacioppo, & Boomsma, 2014). In our study, both self- and parent-loneliness ratings were associated with adult self-reported anxiety and depression outcomes, although higher associations were observed by self-reported loneliness. Self-ratings may be the best indicators of internal traits such as loneliness (Vazire, 2010), but parental reports also pick up on problems that suggest long-term distress. Discrepancies between informants might arise from substantial changes in individuals' social experiences and expectations across adolescent development (Qualter et al., 2015). These differences between self- and parent-report loneliness could also be explained attentional processes in adults, either directly or indirectly (e.g. mothers or fathers) (Cacioppo et al., 2015).

The current study has several limitations. First, this study was representative of a rural area in the Southeast USA but not the US population. Second, loneliness was measured using a series of primary and secondary prompts but only coded into a single item. Insofar as the measure is limited, it may underestimate the effect of loneliness on adult outcomes. Next, this study did not include the potential influence of genetic variation on the young adult's mental health. Genetic and environmental determinants of loneliness (e.g. predispositional vulnerability and exposures to specific life experiences), could contribute to differences across individuals in which pathways operate (Goossens et al., 2015). For example, McGuire & Clifford (2000) showed significant heritability and non-shared environmental influences for children's loneliness using adoption and twin studies (McGuire & Clifford, 2000). The strengths of the present study are large samples, repeated assessments of loneliness across childhood and adolescence, multiple informants, and assessment of a broad spectrum of measured childhood covariates.

Our findings may have implications for future research and clinical practice. First, increased opportunities for social contact and social support, and improved social skills may reduce the risk of future psychiatric disorders in lonely children. However, such study needs to be targeted at the subjective experience of loneliness rather than merely at increasing objective social contacts. Overall, early interventions targeting children's maladaptive social cognitions may be an efficient way to alleviate such subjective feelings of loneliness. Such interventions would have to be implemented in a developmentally appropriate way given the social, cognitive, and emotional changes from childhood to adolescence.

In summary, our findings show that loneliness is relatively common and is observed by parents and children early in life. The question is whether this is a transient dysphoric state that affects current health only or it has the potential to compromise emotional health long term. The current study suggests that loneliness is not transient and the effects are long term. It is also important to remember that even if childhood loneliness is a risk factor for adult distress, most children with childhood loneliness do not experience adult distress. Future research should identify who is at risk for such long-term effects of loneliness and how this risk is propagated across significant developmental transitions.

## Supplementary material

For supplementary material accompanying this paper visit https://doi.org/10.1017/S0033291721001422. click here to view supplementary material
