# Supplementary material for: Childhood loneliness as a specific risk factor for adult psychiatric disorders
Source: Psychol Med. 2021 Jun 14;53(1):227–35. doi: 10.1017/S0033291721001422 (PMC9874978; doi:10.1017/S0033291721001422)
Supplement: Supplementary file 1 [file S0033291721001422sup001.docx]

**Supplementary Figure 1.** Ascertainment of the original Great Smoky Mountains study sample


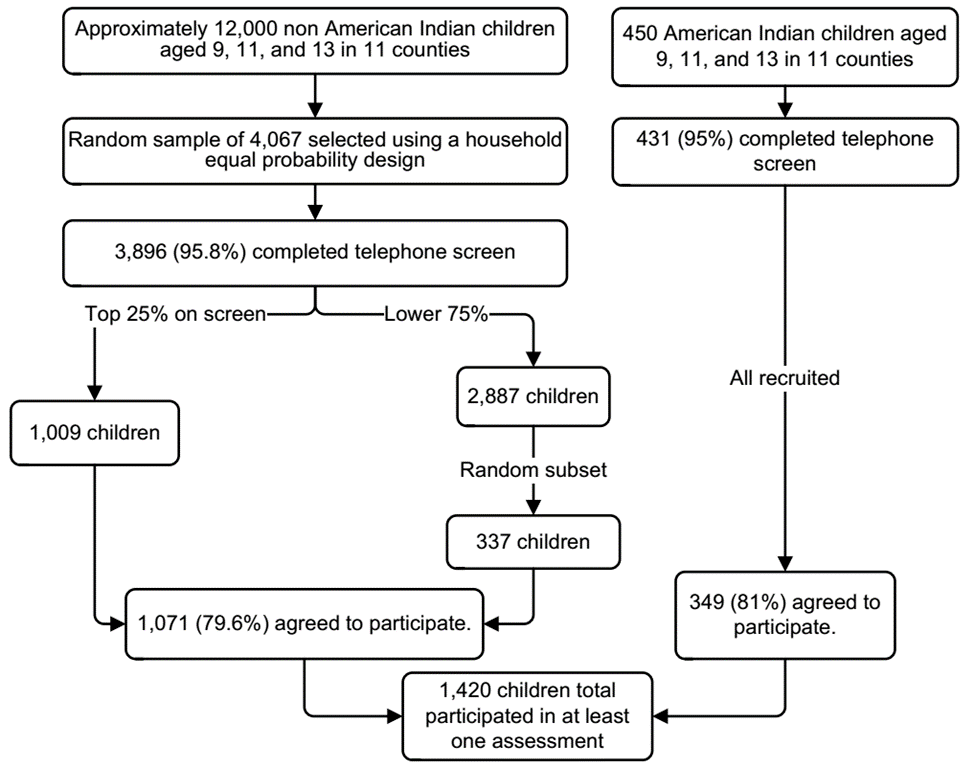


**Supplementary Figure 2.**

**
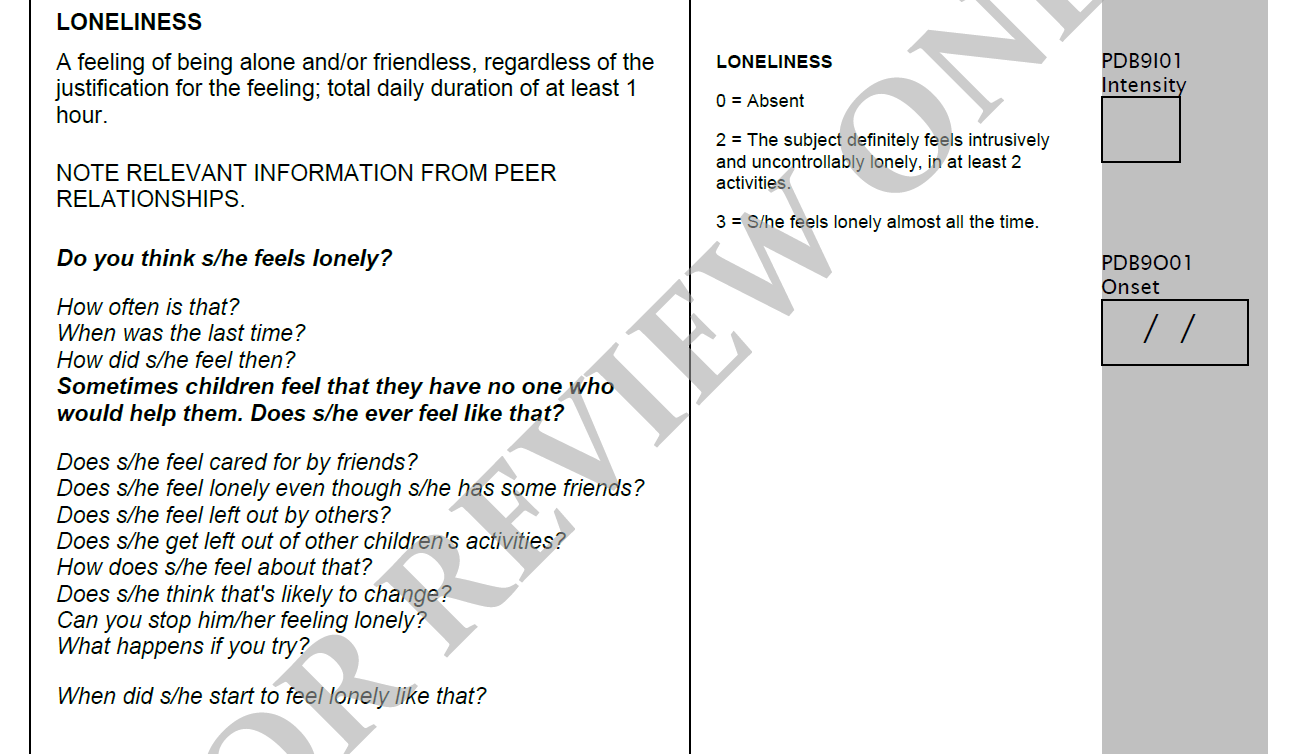
**

**Supplementary Table 1.** Prevalence of childhood loneliness between ages 9 to 16 and

association with childhood peer relationship

|  | Child- and parent-report loneliness | | |
| --- | --- | --- | --- |
|  | *Never lonely*  n (%) | *Ever lonely*  n (%) | *p value ^a^* |
|  |  |  |  |
| *Overall childhood loneliness* | 1230 (86.6) | 190 (13.8%) |  |
| Peer relationship |  |  |  |
| Frequency of contact with peers | 494 (40.2) | 106 (55.8) | 0.081 |
| No Confidant(e) among peers | 712 (57.9) | 116 (61.1) | 0.843 |
| No confidant(e) in family | 685 (57.9) | 116 (61.1) | 0.322 |
| Number of arguments with peers | 104 (8.5) | 48 (25.3) | 0.001 |
| Shyness with peers | 369 (30) | 93 (48.9) | 0.056 |
| Teased or bullied | 372 (30.2) | 115 (60.5) | <0.0001 |

Numbers denotes children included in one or more analyses. Numbers are unweighted, and

percentages are weighted.

^a^*p* value from binary logistic regression of childhood loneliness and childhood peer relationship

outcomes. The models (Odds ratio [ORs]) are adjusted for child sex.

**Supplementary Table 2.** Comparison of latent class growth analysis of

loneliness trajectories model fit indices

|  | Fit indices | | | |
| --- | --- | --- | --- | --- |
|  | Entropy^a^ | AIC^b^ | BIC^c^ | *BLRTp value^d^* |
| Number of class |  |  |  |  |
| 2 | 0.91 | 175.86 | 180.21 | <0.001 |

^a^*AIC = Akaike’s Information Criterion;* ^b^*BIC = Bayesian Information*

*Criterion;* ^c^*BLRT = Bootstrap Likelihood Ratio Test.*
